# Neuroserpin expression during human brain development and in adult brain revealed by immunohistochemistry and single cell RNA sequencing

**DOI:** 10.1111/joa.12931

**Published:** 2019-01-15

**Authors:** Istvan Adorjan, Teadora Tyler, Aparna Bhaduri, Samuel Demharter, Cintia Klaudia Finszter, Maria Bako, Oliver Marcell Sebok, Tomasz J. Nowakowski, Konstantin Khodosevich, Kjeld Møllgård, Arnold R. Kriegstein, Lei Shi, Anna Hoerder‐Suabedissen, Olaf Ansorge, Zoltán Molnár

**Affiliations:** ^1^ Department of Anatomy, Histology and Embryology Semmelweis University Budapest Hungary; ^2^ Department of Physiology, Anatomy and Genetics University of Oxford Oxford UK; ^3^ Neuropathology Unit Nuffield Department of Clinical Neurosciences University of Oxford Oxford UK; ^4^ Department Neurology University of California San Francisco San Francisco CA USA; ^5^ Biotech Research and Innovation Centre University of Copenhagen Copenhagen Denmark; ^6^ Department of Cellular and Molecular Medicine The Panum Institute Faculty of Health Sciences University of Copenhagen Copenhagen Denmark; ^7^ Joint Laboratory for Neuroscience and Innovative Drug Research Jinan University Guangzhou China

**Keywords:** human brain, neurodevelopment, neuroserpin, subplate

## Abstract

Neuroserpin is a serine‐protease inhibitor mainly expressed in the CNS and involved in the inhibition of the proteolytic cascade. Animal models confirmed its neuroprotective role in perinatal hypoxia‐ischaemia and adult stroke. Although neuroserpin may be a potential therapeutic target in the treatment of the aforementioned conditions, there is still no information in the literature on its distribution during human brain development. The present study provides a detailed description of the changing spatiotemporal patterns of neuroserpin focusing on physiological human brain development. Five stages were distinguished within our examined age range which spanned from the 7th gestational week until adulthood. In particular, subplate and deep cortical plate neurons were identified as the main sources of neuroserpin production between the 25th gestational week and the first postnatal month. Our immunohistochemical findings were substantiated by single cell RNA sequencing data showing specific neuronal and glial cell types expressing neuroserpin. The characterization of neuroserpin expression during physiological human brain development is essential for forthcoming studies which will explore its involvement in pathological conditions, such as perinatal hypoxia‐ischaemia and adult stroke in human.

## Introduction

Neuroserpin belongs to the family of serine‐protease inhibitors (Osterwalder et al. 1996; Vivien & Buisson, 2000). It is predominantly expressed in the central nervous system (CNS) (Hastings et al. 1997; Krueger et al. 1997; Teesalu et al. 2004) but recent studies have also revealed extracerebral production (Chéret et al. 2012; Matsuda et al. 2016). Neuroserpin binds tissue plasminogen activator, blocking the conversion of plasminogen to plasmin and thus inhibiting the proteolytic cascade (Vivien & Buisson, 2000; Lee et al. 2017). This function makes neuroserpin a promising therapeutic target in conditions where the proteolytic homeostasis of the CNS is impaired, such as hypoxia‐ischaemia (Millar et al. 2017).

There is still a dearth of knowledge on neuroserpin production and its interaction with molecular pathways in the human brain. This greatly hinders the translation of its already discovered neuroprotective roles in animal models (Yepes et al. 2000; Zhang et al. 2002; Lebeurrier et al. 2005; Ma et al. 2012; Li et al. 2017) towards developing pharmacological strategies and eventually bedside treatment. Our work aims to lay the foundations for future studies by discovering the spatiotemporal expression patterns of neuroserpin during physiological human brain development.

The current paper focuses on the pallial part of the developing telencephalon. Our special region of interest was the subplate, a transient developmental structure subjacent to the cortical plate. The precise role of this layer is still largely unknown, but there are data suggesting its important function in directing neuronal migration, the ingrowth of thalamocortical axons and the formation of associative corticocortical connections (Kostovic & Rakic, 1980, 1990; Kostovic & Judas, 2002). Some of the earliest synapses are formed in this layer during cortical development (Kostović & Judas, 2007). Moreover, the subplate represents the main efferent output from the early midfoetal cortex since development of cortical plate efferents is somewhat delayed and they have not yet reached the pons, medulla oblongata (Ip et al. 2011) or the spinal cord (Eyre et al. 2000). During the ingrowth of thalamocortical afferents to the cortical plate, these fibers are accumulated in the subplate (Krsnik et al. 2017). The thalamocortical projections have a complex interplay with the subplate and establish transient circuits during this period, once considered the ‘waiting’ period. The subplate has a very unique ECM composition which is particularly abundant in acidic glycosaccharides and therefore can be visualised by PAS‐Alcian Blue staining (Kostovic & Rakic, 1990), which was also utilised in our study.

In this study, we identified stages of neuroserpin expression based on layer‐ and cell type‐ specific immunohistochemical detection of neuroserpin in the developing human cortical plate and subplate. These results were corroborated by single cell RNA sequencing data confirming the neuronal and glial production of neuroserpin (*Serpini1*) and revealing changing co‐expression patterns between younger (6–15th gestational week) and older (15–22nd gestational week) cases.

In the first part of this paper, we describe the detailed distribution pattern of neuroserpin immunoreactive cells based on immunohistochemistry. In the second part, we provide an in‐depth analysis of *Serpini1* co‐expression networks and specify the clusters responsible for its production.

## Methods

### Subjects

Anonymised cases were received from the Oxford Brain Bank (OBB), the Netherlands Brain Bank (NBB) and the hGENESIS collection, Department of Cellular and Molecular Medicine, Faculty of Health and Medical Sciences, University of Copenhagen. All material has been collected from donors from whom written informed consent had been obtained by the OBB or NBB for brain autopsy and use of material and clinical information for research purposes. We included samples in the study with available clinical information and postmortem neuropathological diagnoses confirming minimal hypoxic insults in the CNS. The identifier and age of cases are shown in Table 1. Prenatal ages were described using gestational weeks, which refer to the date of the last menstruation bleeding occurring before pregnancy. This usually precedes conception by 2 weeks (Bannister et al. 1995).

**Table 1:** List of cases included in the immunohistochemical analysis

Identifier	Age	Source
Z3379/124	7 gw	hGENESIS
1317/196	9 gw	hGENESIS
1425/270	10 gw	hGENESIS
1265/94	13 gw	OBB
146/11	14 gw	OBB
1248/94	16 gw	OBB
1120/95	16 gw	OBB
41/14	18 gw	OBB
B5349	19 gw	OBB
153/12	19 gw	OBB
121/5	19 gw	OBB
155/10	21 gw	OBB
85/06	21 gw	OBB
65/08	22 gw	OBB
59/07	25 gw	OBB
57/09	25 gw	OBB
193/04	25 gw	OBB
144/12	30 gw	OBB
72/14	33 gw	OBB
1054/91	38 gw	OBB
C2196	38 gw	OBB
C3492	40 gw	OBB
C2450	3 pw	OBB
C1951	3 pw	OBB
C3175	4 pw	OBB
24/14	3 pm	OBB
143/13	1 year	OBB
77/13	1 year	OBB
105/15	1.5 years	OBB
1/15	2 years	OBB
86/15	7 years	OBB
S11/081	55 years	NBB
S12/002	55 years	NBB
S12/071	57 years	NBB
S10/196	60 years	NBB
S11/096	70 years	NBB
S12/059	78 years	NBB

### Immunohistochemistry

Serial sections (6 μm thick) were cut in the coronal plane from paraffin‐embedded blocks containing the frontal and temporal cortices and mounted on slides. Our immunohistochemical analysis was done as described in detail in an earlier study (Adorjan et al. 2017). Briefly, the following four primary anti‐neuroserpin antibodies were applied: (1) rabbit, Abcam ab33077, 1 : 200; (2) mouse, Abcam ab55587, 1 : 200; (3) rabbit, Sigma HPA 001565, 1 : 200; (4) rabbit, Sino Biological 11107‐RP02, 1 : 500. Application on adjacent (serial) sections gave similar staining patterns. In order to recognise the layers in developing cortex, staining with Nissl and PAS‐Alcian Blue were done, as described previously (Kostovic et al. 2002).

### Image analysis

Slides were digitised using slidescanners (AperioScanScope AT Turbo, Leica Biosystems; 3DHistech) at 20× and 40× magnification and stored on a server (msdlt‐slide.dpag.ox.ac.uk). The regions of interest were outlined using the imagescope programme (Aperio, v11.2.0.780) and the longest diameter of every neuroserpin‐immunopositive (neuroserpin‐ip) cell body in the telencephalic wall was manually measured. Heatmaps were visualised based on the coordinates of neuroserpin‐ip cells using qgis 3.2.2 software (Ic6 line).

### Single cell RNA sequencing in developing human

Single cell sequencing was performed and analysed as described in Nowakowski et al. (2017). In addition to the analyses provided in that paper, we performed analyses of *Serpini1* (neuroserpin) across ages and cell types to identify populations with enriched expression. To do this, we performed correlations and violin plots from the normalised sequencing read counts per million with the metadata properties of interest. The threshold for considering a gene to be expressed was a count per million greater than 0 in at least 30 cells in the dataset. A count of transcripts per million of 2 lies in the bottom quartile of expression for all genes.

### Single cell RNA sequencing data analysis in adult human

Violin plots for *Serpini1* expression were generated from human cortex single nucleus dataset available in Lake et al. (2016). First, neuronal nuclei from the temporal cortex were isolated from the whole dataset, resulting in 1406 single nucleus transcriptomes. Classification of neuronal clusters into excitatory (Ex) and inhibitory (In) subtypes was done as in Lake et al. (2016). The level of gene expression was estimated using transcript per million mapped reads (tpm), and the threshold of tpm ≥ 10 was used for *Serpini1* to be considered expressed.

## Results

Our immunohistochemical analysis, which spanned from the 7th gestational week (gw) until adulthood and was based on 37 cases, revealed five different patterns in terms of the distribution of neuroserpin immunoreactive cells in the developing human cortex:

*Neuroserpin begins to be produced in the first trimester (7–10th gw)*

**Figure 1:**
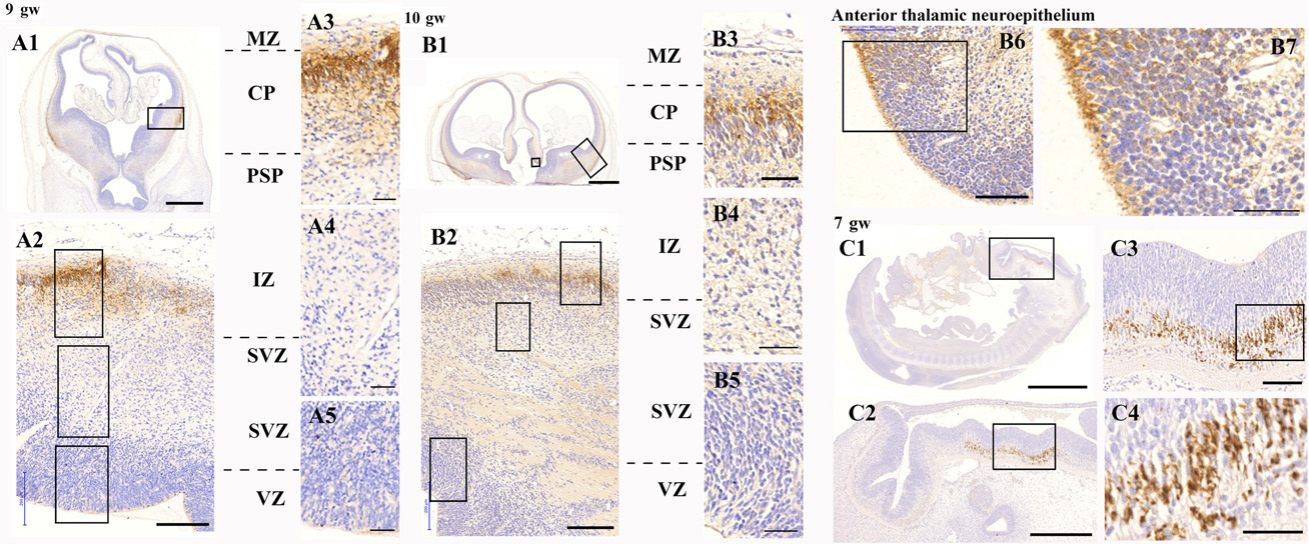
Neuroserpin expression in the first trimester of human brain development. A1–A5: neuroserpin is expressed in the upper cortical plate of the ventrolateral pallium in the 9th gw. B1–B5: neuroserpin is expressed in the upper cortical plate of the ventrolateral pallium in the 10th gw. B6–B7: the anterior thalamic neuroepithelium also contained neuroserpin‐ip cells with long processes. C1–C4: neuroserpin is expressed by a distinct neuronal population in the rhombencephalon in the 7th gw. No telencephalic expression was observed at this stage of development. MZ, marginal zone; CP, cortical plate; PSP, pre‐subplate; IZ, intermediate zone; SVZ, subventricular zone; VZ, ventricular zone. Scale bars: (A1) 1000 μm, (A2, B2) 200 μm, (A3–A5) B3–B6, (C3) 100 μm, (B1, C1) 2000 μm, (B7, C4) 50 μm, (C2) 500 μm.

Neuroserpin was expressed in the telencephalon as early as the 9th gw (Fig. 1A). Neuroserpin immunoreactivity was localised in cells with elongated morphology and cell processes in the upper part of the developing cortical plate adjacent to the marginal zone (for a detailed description of lamination see Kostović & Judas, 2007). The immunopositive (ip) cells were restricted to the developing ventrolateral pallium. No ip cells were seen in the developing diencephalon, mesencephalon or rhombencephalon at this stage. The aforementioned neuroserpin‐ip cell population in the ventrolateral pallium was also present in the 10th gw (Fig. 1B). Besides, neuroserpin‐ip perikarya and processes appeared in the ventricular zone of the anterior thalamic neuroepithelium (Fig. 1B). Although no telencephalic expression was detected in the 7th gw, there was a conspicuous neuroserpin‐ip population situated in the rhombencephalon (Fig. 1C).

*The early second trimester (13th–16th gw)*Neuroserpin immunoreactivity was localised in migrating neurons through the whole cortical plate and subplate.

**Figure 2:**
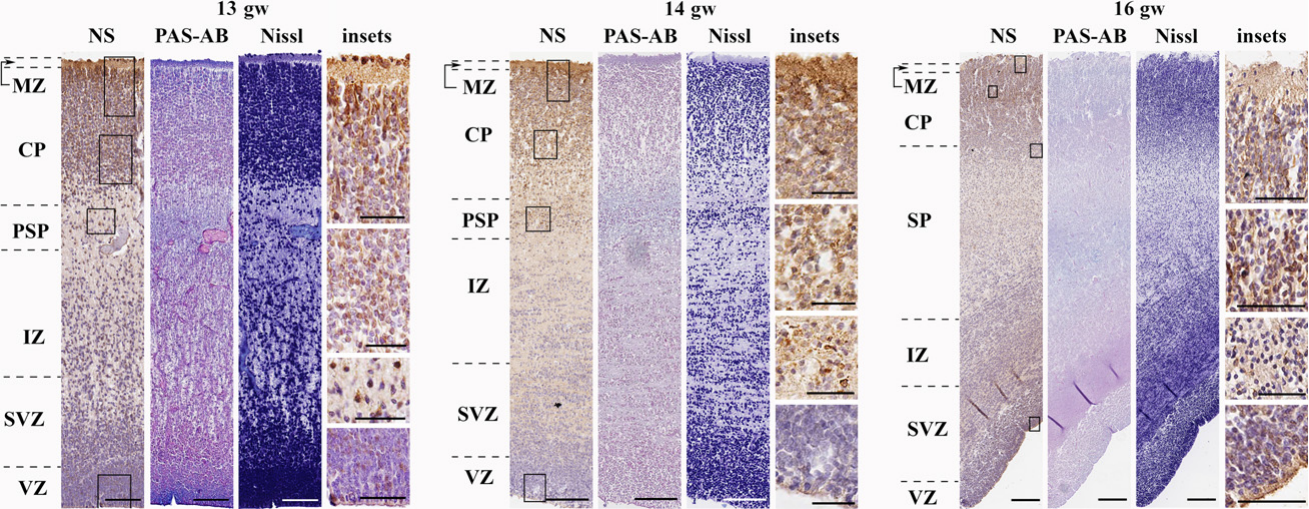
Neuroserpin expression in the early second trimester (frontal lobe). Neuroserpin‐ip cells were present through the cortical plate and subplate. They had long processes and elongated perikarya showing migratory morphology. Minimal expression was seen in the marginal zone. Neuroserpin‐ip cells were observed in the ventricular zone of the lateral and caudal ganglionic eminences. MZ, marginal zone; CP, cortical plate; PSP, pre‐subplate; SP, subplate; IZ, intermediate zone; SVZ, subventricular zone; VZ, ventricular zone. Scale bars: (13th gw) NS, PAS‐AB, Nissl: 100 μm, insets: 50 μm; (14th–16th gw) NS, PAS‐AB, Nissl: 200 μm, insets: 50 μm.

By the second trimester, neuroserpin immunoreactivity was localised in cortical plate and subplate cells with long processes showing migratory morphology (Fig. 2). These were present through the whole thickness of the cortical plate. Production in the ventricular zone, however, showed regional differences. In general, cortical, LGE and CGE ventricular zones contained neuroserpin‐ip cells, whereas MGE did not (data not shown).

*The middle of the second trimester (18th–22nd gw)*Neuroserpin immunoreactivity was localised in migrating/maturing neurons of the middle and deep cortical plate and subplate.

**Figure 3:**
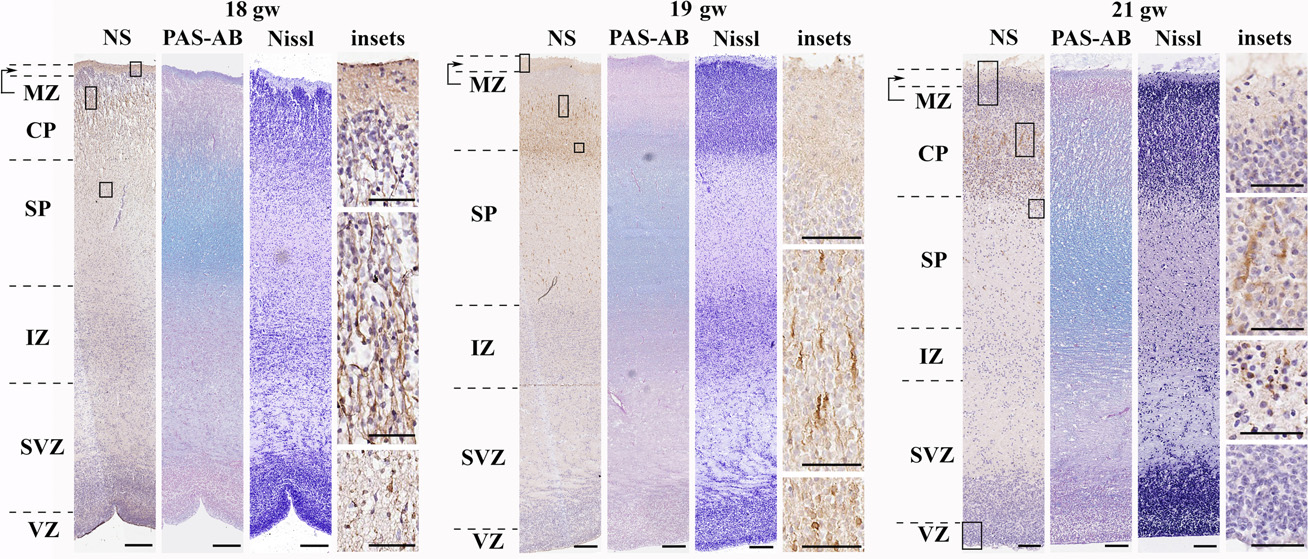
Neuroserpin expression in the middle second trimester in the human telencephalon (frontal lobe). Neuroserpin‐ip cells with elongated perikarya and long processes directed to the pial surface were mainly seen in the middle and deep cortical plate and subplate. No neuroserpin‐ip cells were observed in the upper cortical plate. MZ, marginal zone; CP, cortical plate; SP, subplate; IZ, intermediate zone; SVZ, subventricular zone; VZ, ventricular zone. Scale bars: (18th gw) NS, PAS‐AB, Nissl: 100 μm; (19th–21st gw) NS, PAS‐AB, Nissl: 200 μm, insets: 50 μm.

During the middle of the second trimester, neuroserpin immunoreactivity was localised to cortical and subplate cells with migratory morphology, suggesting its presence mostly in migrating neurons (Fig. 3). In contrast to the previous stage, neuroserpin‐ip cells were distributed in the middle and deeper cortical plate but not in the upper cortical plate. Interregional differences were seen in case of the VZ, as neuroserpin‐ip cells were usually contained in dorsal regions but not ventral ones (data not shown). No neuroserpin immunoreactivity was seen in the VZ from the 21^st^ gw (Fig. 3).

*From the late second trimester until the end of the first postnatal month (25th gw–1st pm)*Neuroserpin was found in migrating/maturing neurons of the deep cortical plate and subplate.

**Figure 4:**
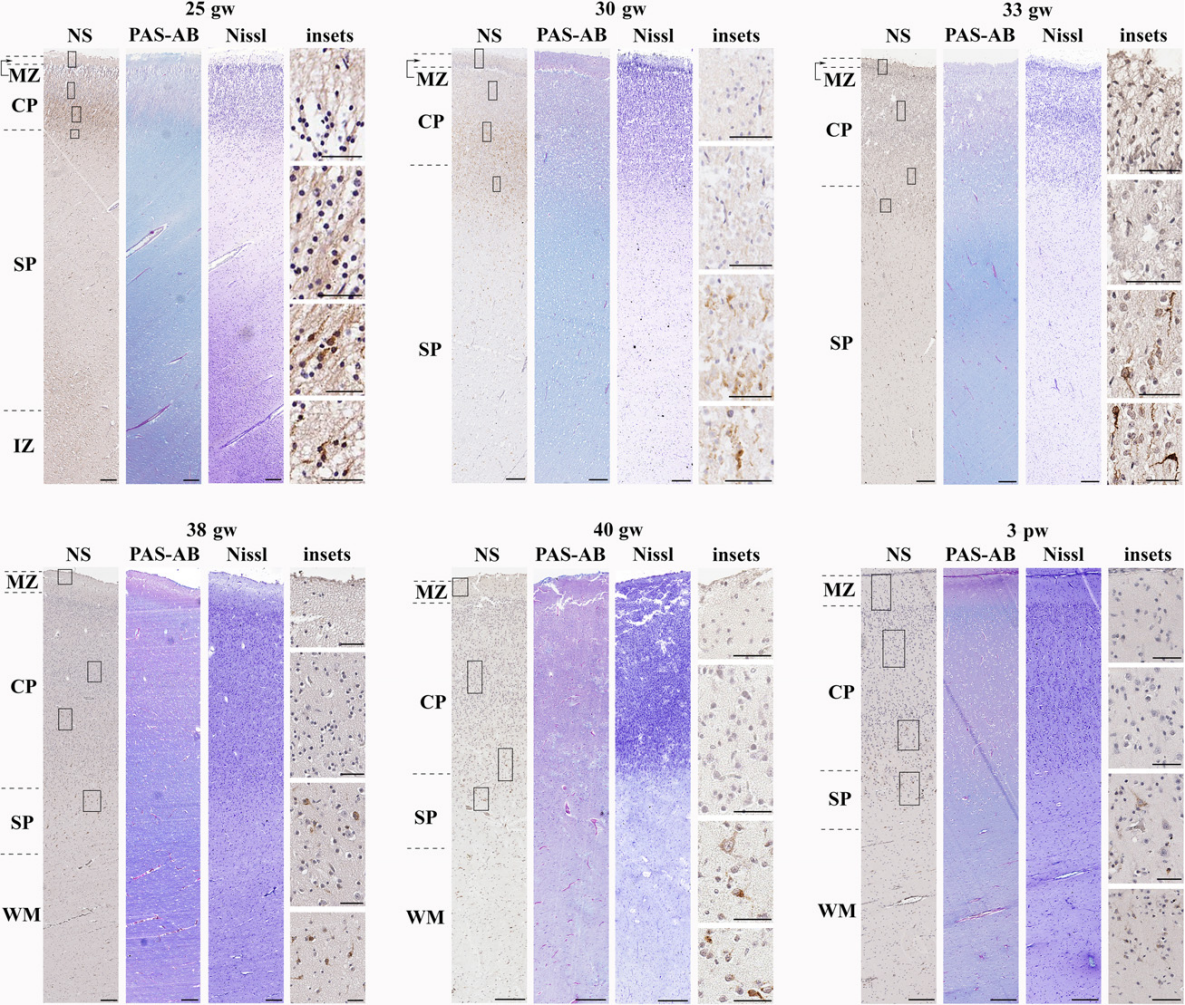
Neuroserpin expression from the late second trimester until the first postnatal month (frontal lobe). Neuroserpin‐ip cells were situated to the deep cortical plate and subplate with a pyramidal and elongated shape. The upper and middle regions of the cortical plate were devoid of neuroserpin immunoreactivity. MZ, marginal zone; CP, cortical plate; SP, subplate; IZ, intermediate zone; WM, white matter. Scale bars: (25th gw–3rd pw) NS, PAS‐AB, Nissl: 200 μm, insets: 50 μm.

During the late second trimester and the whole third trimester, neuroserpin‐ip cells were localised to the deep cortical plate and subplate (Fig. 4). Their morphology suggested migrating and maturing neurons, predominantly of pyramidal type. This pattern remained characteristic of neuroserpin expression until the first postnatal month. The VZ was devoid of neuroserpin‐ip cells at this stage. Their presence in the upper and middle cortical plate was minimal (Fig. 4).

*From the third postnatal month until adulthood (3rd pm‐adult)*

**Figure 5:**
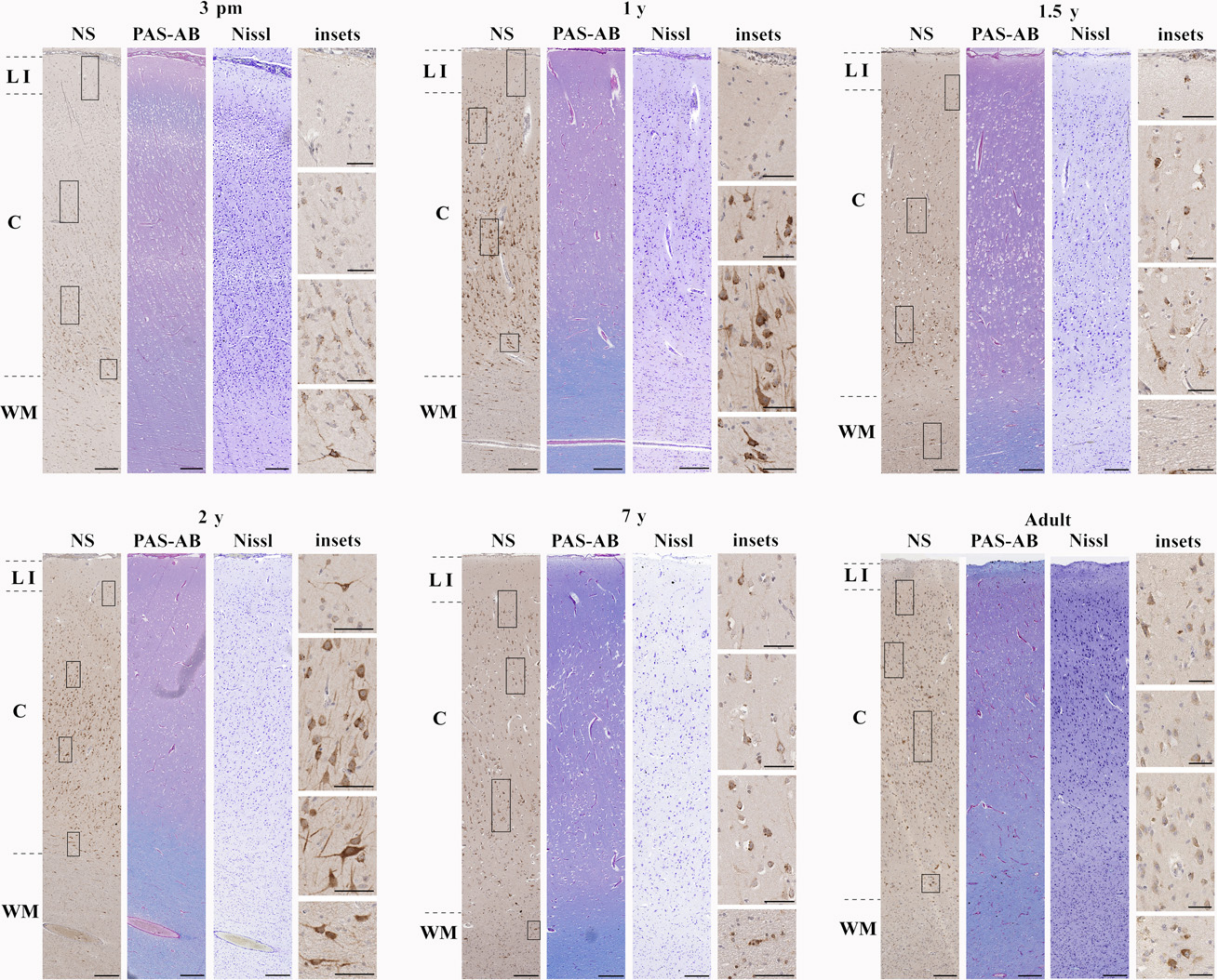
Neuroserpin expression from the third postnatal month until adulthood in the cortex (frontal lobe). Neuroserpin‐ip neurons were observed throughout layers of the cortex with the exception of layer 1, where minimal immunoreactivity was present. The majority of neuroserpin‐ip neurons had a pyramidal morphology. LI, layer 1; C, cortical layers 2–6; WM, white matter. Scale bars: (3 pm–adult) NS, PAS‐AB, Nissl: 200 μm, insets: 50 μm.

After the 3rd postnatal month, the expression of neuroserpin was propagated towards the upper cortical layers (Fig. 5). By the second year of age, all cortical layers consisted of neuroserpin‐ip cells, most of them with pyramidal morphology. This pattern remained characteristic of childhood and adulthood as well.

**Figure 6:**
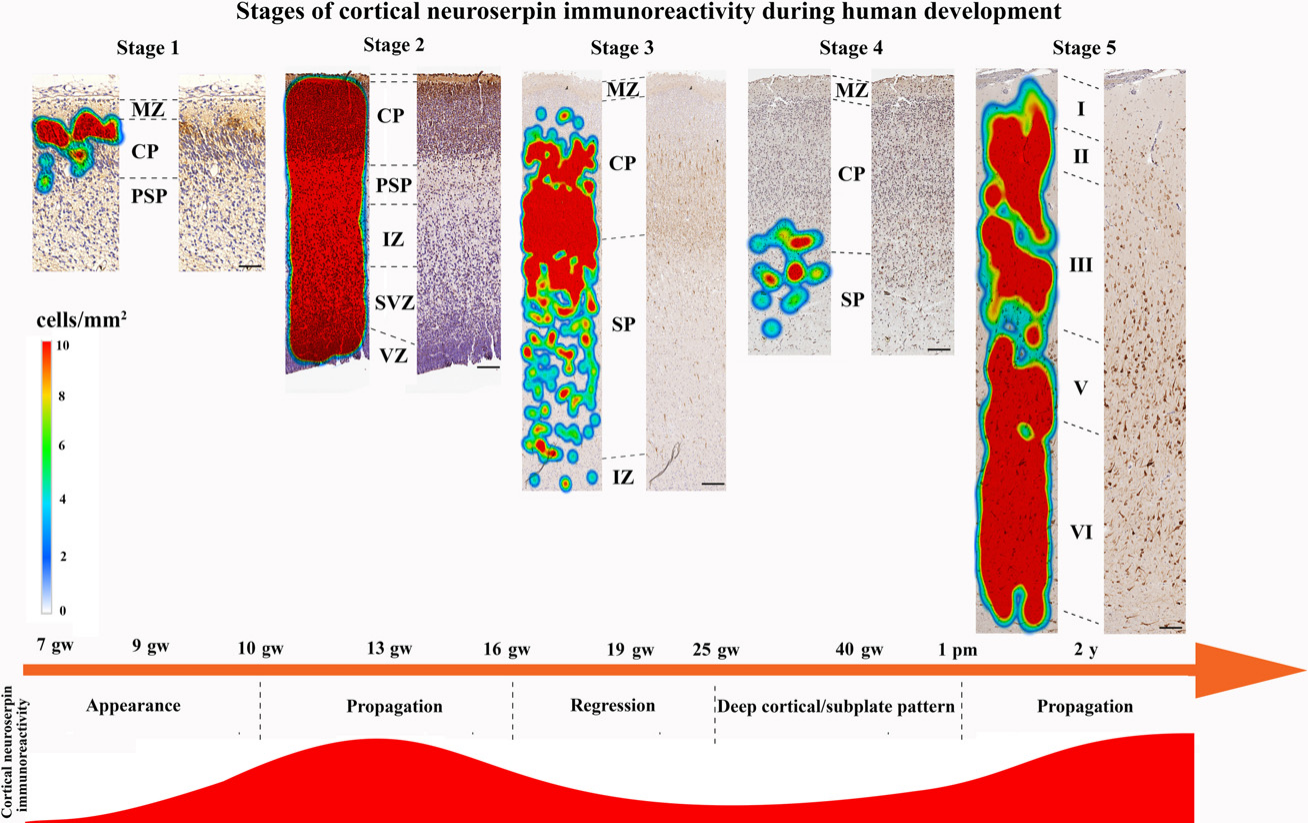
Heatmaps showing the characteristic stages of neuroserpin immunoreactivity in the developing human cortex based on the density of neuroserpin‐ip cells. MZ, marginal zone; CP, cortical plate; PSP, pre‐subplate; IZ, intermediate zone; SVZ, subventricular zone; VZ, ventricular zone. Scale bars: (stage 1) 50 μm, (stage 2) 80 μm, (stage 3) 130 μm, (stage 4) 140 μm, (stage 5) 150 μm.

Heatmaps based on the co‐ordinates and density of neuroserpin‐ip cells helped to distinguish the distribution patterns of these cells and identify the above‐described five characteristic stages during human cortical development (Fig. 6 and Supporting Information Fig. S1).

*Single cell RNA sequencing in embryonic brain revealed pyramidal neurons and migrating neurons as the main sources of* Serpini1

**Figure 7:**
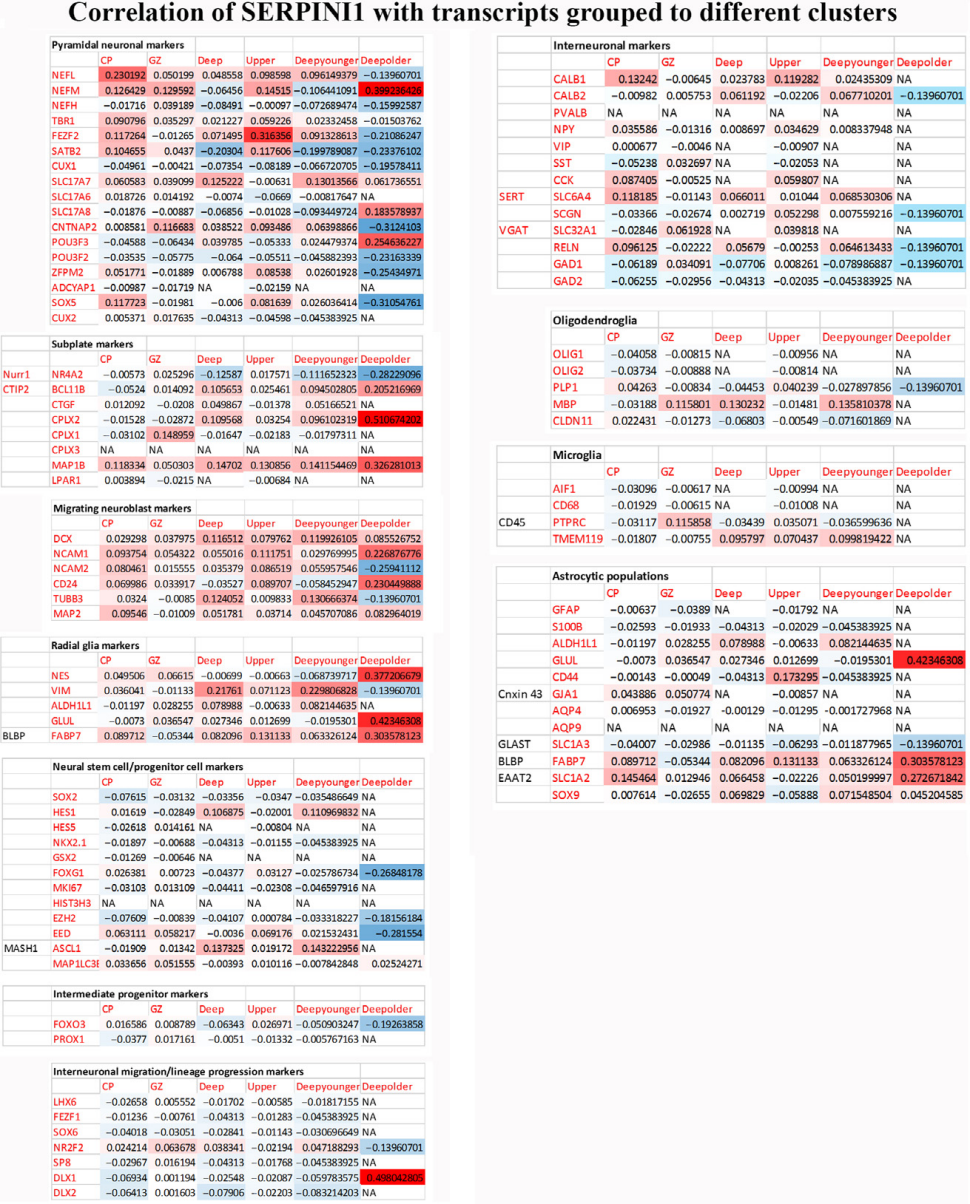
Correlation levels of *Serpini1* to different transcripts during development visualised by heatmap (based on Nowakowski et al. 2017). Transcripts are grouped in specific cell types. CP, cortical plate; GZ, granular zone; Deep, deep cortical; Upper, upper cortical; Deep younger, deep cortical <15 gestational week; Deep older, deep cortical > 15th gw.

Single cell RNA sequencing made possible the detailed cell‐type analysis of neuroserpin‐expressing cells. This work was based on correlation levels of *Serpini1* to more than 20 000 other transcripts within a dataset already clustered to different cell types during human brain development (Nowakowski et al. 2017). This approach confirmed *Serpini1* expression in post migratory pyramidal neurons and migrating neurons, and the scarcity of neuroserpin production by neural stem/progenitor cells, oligodendrocytes and microglia (Fig. 7). Also, our results on co‐expression patterns highlighted the possible astrocytic‐ (*Glul, Fabp7, Slc1a2*) and subplate‐specific (*Bcl11b, Cplx2, Map1b*) cell‐type origin of neuroserpin production (Fig. 7).

**Figure 8:**
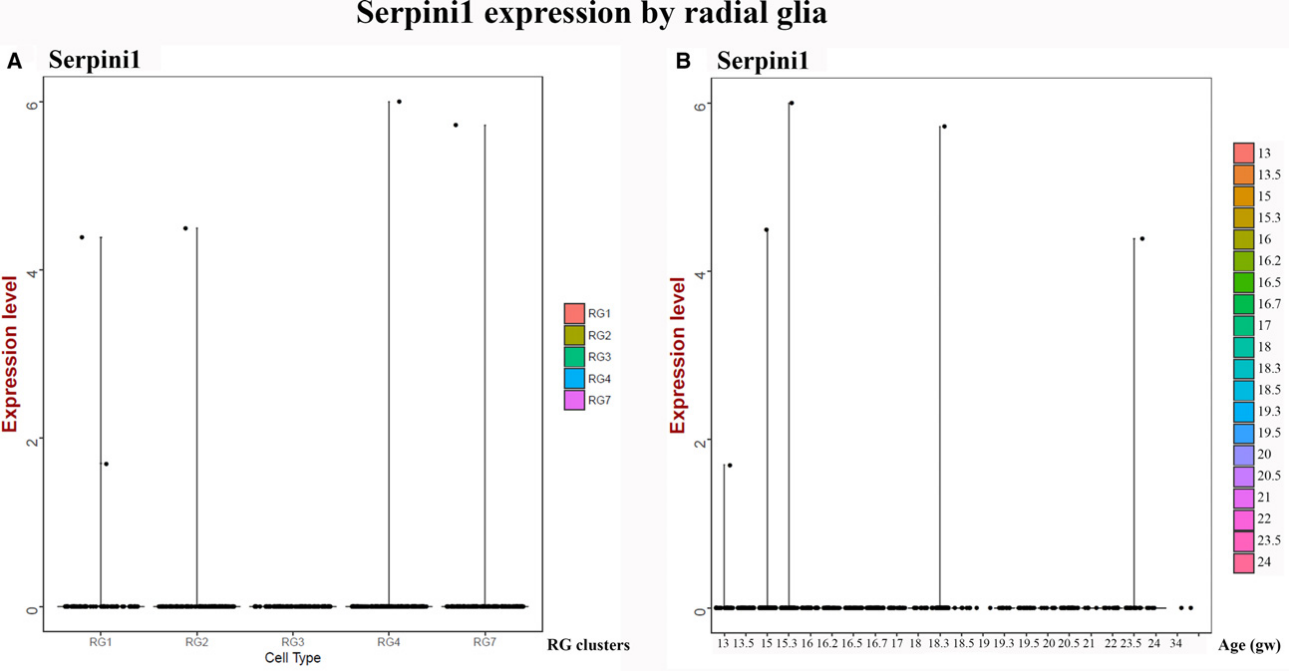
Expression of neuroserpin in developing human cortex described by single cell transcriptomics analysis. (A) Violin plot for neuroserpin expression in different radial glial subtypes (counts per million). (B) Violin plot for neuroserpin expression by radial glial clusters plotted along the age (gestational week) (Nowakowski et al. 2017).

According to our data, *Serpini1* was expressed in 11.37% of total neurons (170/1494 across all developmental ages examined. Almost half of them (36.47%, 62/170) had acquired the excitatory neuron‐specific neurofilament *Nefm*. Only a handful of them belonged to the calbindin‐ or calretinin‐interneurons (2.35% and 2.35%, 4/170 and 4/170, respectively). Radial glial clusters were identified as an early source of low levels of *Serpini1* expression that peaked around the 15^th^ gw (Fig. 8). However, no robust neuroserpin‐ip radial glial pattern was observed by our immunohistochemical analysis (Figs 1, 2, 3).

*In adult cortex, neuroserpin is ubiquitously expressed in pyramidal neurons and GABAergic interneurons*

**Figure 9:**
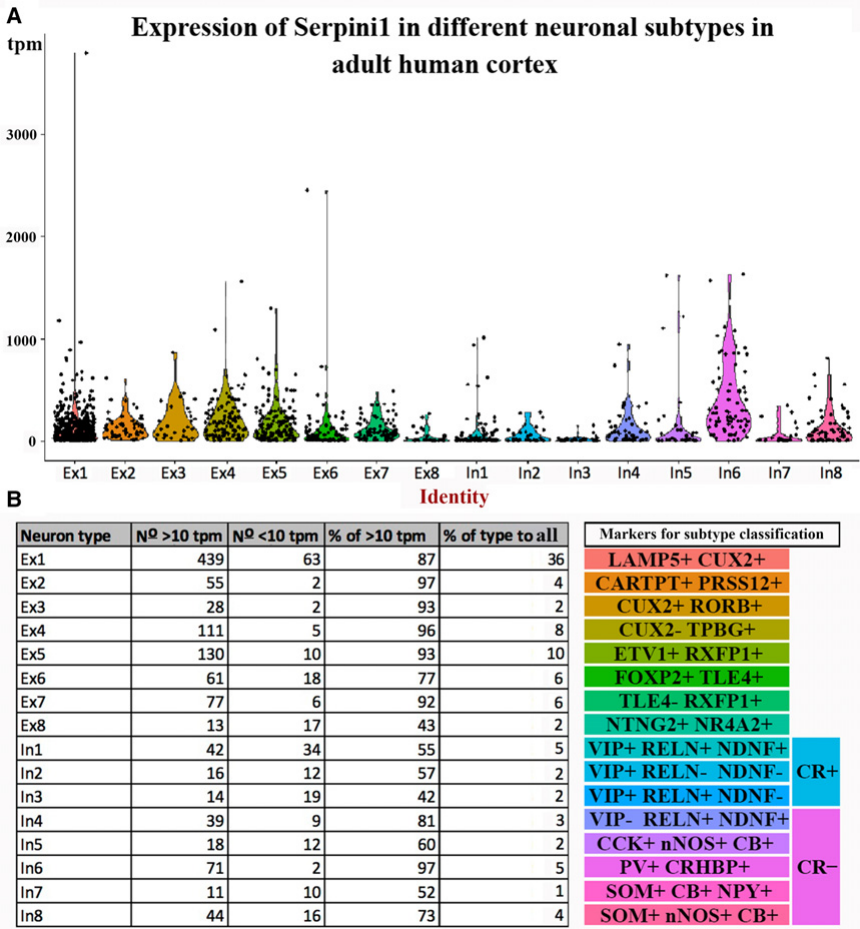
Expression of neuroserpin in adult human temporal cortex as identified by single cell transcriptomics analysis. (A) Violin plot for neuroserpin expression in each subtype of excitatory and inhibitory neurons from dataset of single cell RNA sequencing data in Lake et al. (2016). (B) Table summarizing the number and percentage of neurons for each subtype that expressed neuroserpin above the threshold (tpm > 10). Subtype‐markers are shown on the right hand side.

To describe expression pattern of *Serpini1* gene across neuronal subtypes in the adult human cortex, we utilised a previously published dataset of the temporal cortex (Lake et al. 2016). Of 1406 excitatory and inhibitory neurons sequenced, > 80% of neurons expressed *Serpini1* with tpm > 10 that was set as a threshold of expression (Fig. 9A,B). All subtypes of pyramidal neurons except deep layer Ex6 and Ex8 had ~ 90% or more neurons expressing *Serpini1*. On average, ~50% of GABAergic interneurons expressed *Serpini1* above the threshold. However, a few groups of GABAergic interneurons expressed higher levels of *Serpini1*; in particular, almost 100% of In6 cluster that can be assigned as parvalbumin‐positive interneurons expressed *Serpini1*. Among other interneurons expressing higher levels of *Serpini1* are In4 (reelin and Ndnf‐double positive, but vasoactive intestinal peptide‐negative interneurons) and In8 (somatostatin and Nos1‐double positive interneurons).

## Discussion

The regulation of the proteolytic homeostasis by neuroserpin in the human brain may be of crucial importance in the reaction to hypoxia‐ischaemia (Millar et al. 2017). To understand its role in brain pathology, however, first its tissue distribution and cell type‐specific production should be thoroughly analysed using various methods. Our study revealed hitherto unknown patterns of neuroserpin production during human brain development.

The importance of our work is underscored by the fact that there is a scarcity of descriptive morphological studies done in human. These works have been eclipsed by the boom of molecular biological approaches and the advent of big data in recent decades (Hawrylycz et al. 2015; Dillman et al. 2017). However, without descriptive morphological studies in human there is no prospect of linking molecular biological data to distinct neural circuits and eventually understanding the function of the investigated molecules in a spatial‐dependent context.

The first telencephalic expression of neuroserpin was observed in the 9th gw when the developing pallium is formed as a trilaminar structure (marginal zone, cortical plate and pre‐subplate, Kostović & Judas, 2007). Based on the morphology of neuroserpin‐ip cells, most likely they belong to migrating neurons reaching the interface between the marginal zone and cortical plate. No neuroserpin‐ip cells were seen in the deeper pallial layers or in the ventricular zone at this early stage of brain development.

The expansion of neuroserpin‐ip cells throughout layers of the telencephalic wall was observed between the 13rd and 18th gw. The identity of these cells as migrating neurons and maturing excitatory neurons was confirmed by single cell RNA sequencing data. This period coincides with the first major wave of neurons migrating from the germinal zones towards the cortical plate. As the telencephalic wall increased in thickness during the late second trimester, neuroserpin‐ip cells gradually localised to deeper layers of the developing cortical plate and the subplate. This matched with single cell RNA sequencing data that characterised a novel neuroserpin‐ip subplate cell type co‐expressing *Cplx2*,* Bcl11b (Ctip2)* and *Map1b*. *Map1b* has already been predicted as a potential subplate marker in rat (Teng et al. 2001).

The selective expression of neuroserpin by deep cortical plate/subplate cells during the third trimester and early postnatal period (between the 25^th^ gw and 1^st^ postnatal month) suggests dynamically changing functions of this molecule, perhaps adapting to the need of the lamination sequence, thalamocortical fibre ingrowth into the cortical plate and maturation of cortical plate circuits. Our observations imply that neuroserpin expression is down‐regulated in migrating neurons after passing the subplate/deep cortical plate. A question arises why this process may be necessary because such a down‐regulation may cause vulnerability to hypoxic stress. However, increased vulnerability of early cortical plate neurons may be within the scope of the developmental programme, as this phenomenon may assist the selection of the fittest neurons within the cortical plate. Those neurons which do not fit properly into the neural circuitry, i.e. those with impaired firing patterns/metabolic activity, do not survive and may undergo apoptosis and be phagocytosed by microglia (Harry & Kraft, 2012). The continuous expression of neuroserpin by deep cortical/subplate neurons and hence their selective resistance to hypoxic stress may be necessary because during the inside–out lamination sequence these neurons provide the directing cues for incoming waves of migrating neurons. Therefore it is important to have these neurons in place and not replaced by newcomers.

It is important to note that other factors may also contribute to the vulnerability of cortical plate/migrating neurons to hypoxia in the early stage of development (which coincides with the first propagation of neuroserpin expression). For example, the absence of glutamatergic input on these early cortical plate/migrating neurons can also lead to hypoxic vulnerability, as explained by the lack of NMDA receptor‐mediated neuroprotection (Marini et al. 2001; Marini et al. 1998).

The propagation of neuroserpin expression after the 3^rd^ postnatal month signals the end of the time‐window when cortical plate neurons may be particularly vulnerable to hypoxic stress. Those neurons which were properly fitted into the neural circuitry and survived this selection period, may acquire the neuroprotective protease inhibitor, neuroserpin. The role of neuroserpin in adult cortex is underscored by its ubiquitous expression within almost all neuronal subtypes shown in this study and others (Teesalu et al. 2004). Because of the short half‐life of this molecule (~ 10 min, Lee et al. 2017) its constitutive expression may be vital in blocking the activation of the proteolytic cascade in the CNS.

Results from developmental animal models reported the subplate specific expression of neuroserpin (mouse: Kondo et al. 2015). Nonetheless, caution is warranted when extrapolating data from rodent models to human because the deep cortical expression pattern observed in adult mouse (Wu et al. 2010; Kondo et al. 2015) is markedly different from that found in adult human. The almost pan‐neuronal neuroserpin expression in adult human cortex (Teesalu et al. 2004) is confirmed by our study, which suggests different roles of this molecule or increased anti‐proteolytic demand by neurons in human. Future studies should reveal whether the pan‐neuronal cortical expression of neuroserpin is a unique feature in human or other primate taxa, and should decipher the possibility of changing functions of neuroserpin between different mammalian taxonomic groups.

Our work provides cues for the possible proteolytic mechanism involved in cortical plate development, especially for a selection period for cortical plate neurons which may coincide with the regression of neuroserpin from the cortical plate starting from the 18th gw. This period extends until the propagation of neuroserpin within the cortex starting from the 3rd postnatal month. The endogenous low level of neuroserpin expression observed in cortical plate neurons during the perinatal period may be promising for therapeutic approaches which aim to improve neuronal survival by increasing the baseline level of neuroserpin following perinatal hypoxia‐ischaemia. Future studies are warranted to reveal the role of neuroserpin in human perinatal hypoxia and other relevant models (neuroserpin KO mouse, Rice‐Vannucci) focusing on molecular interplays with hypoxia/proteolysis and synaptic assembly networks.

## Conflict of interest

The authors have no conflicts of interest to declare.

## Author contributions

I.A. and Z.M. conceived the study. I.A., T.T. and K.M. did the immunohistochemical staining. I.A., T.T. and C.F. scanned sections and measured immunoreactivity. B.M. performed Nissl and PAS‐Alcian Blue staining. S.M. and T.T. carried out the heatmap analysis. B.A., T.J.N. and A.R.K. provided single cell RNA sequencing data on developing human brain. K.K. and S.D. carried out single cell RNA sequencing data analysis on adult human dataset. All authors interpreted the data, and contributed to and approved the final version of the manuscript.

## Supporting information


**Fig. S1.** Heatmaps showing the distribution of neuroserpin immunoreactivity during stage 2 (13th gw) from Fig. 6.
