# Supplementary figures and images for: Neuroserpin expression during human brain development and in adult brain revealed by immunohistochemistry and single cell RNA sequencing
Source: J Anat. 2019 Jan 15;235(3):543–54. doi: 10.1111/joa.12931 (PMC6704272; doi:10.1111/joa.12931)

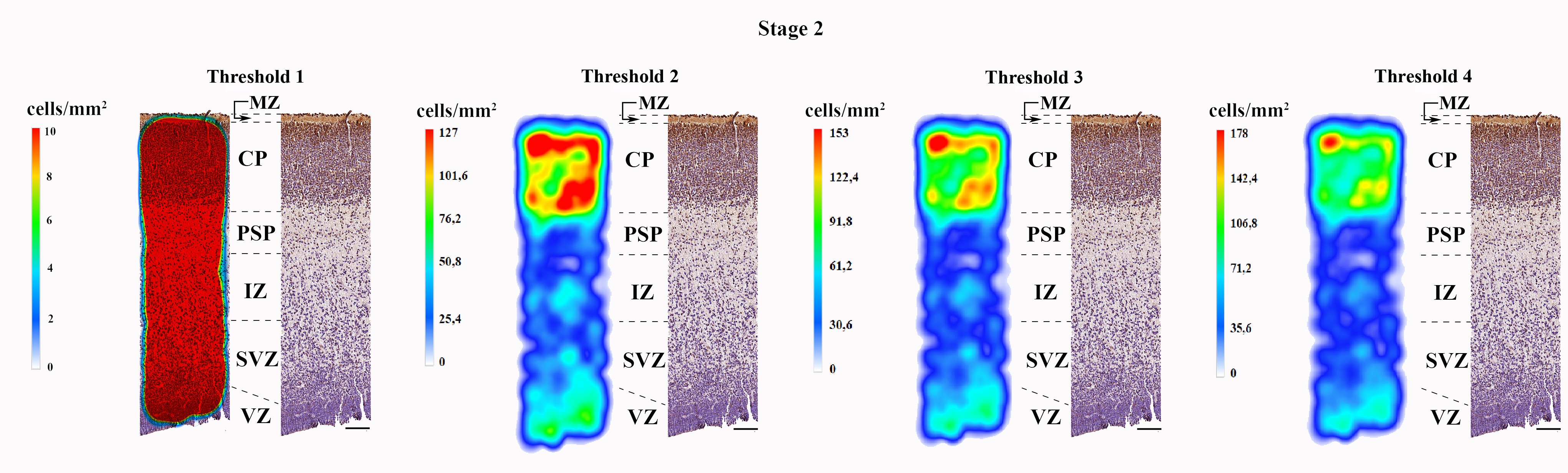

Supplement: Supplementary file 1 — Fig. S1. Heatmaps showing the distribution of neuroserpin immunoreactivity during stage 2 (13th gw) from Fig. 6. [file JOA-235-543-s001.tif]
